# Alginate Gel Encapsulated with Enzybiotics Cocktail Is Effective against Multispecies Biofilms

**DOI:** 10.3390/gels10010060

**Published:** 2024-01-14

**Authors:** Daria V. Vasina, Nataliia P. Antonova, Elena V. Shidlovskaya, Nadezhda A. Kuznetsova, Alexander V. Grishin, Elizaveta A. Akoulina, Ekaterina A. Trusova, Anastasiya M. Lendel, Elena P. Mazunina, Sofia R. Kozlova, Andrei A. Dudun, Anton P. Bonartsev, Vladimir G. Lunin, Vladimir A. Gushchin

**Affiliations:** 1 N.F. Gamaleya National Research Centre for Epidemiology and Microbiology, Ministry of Health of the Russian Federation, 123098 Moscow, Russia; northernnatalia@gmail.com (N.P.A.); lenitsa@gmail.com (E.V.S.); nadyakuznetsova0@gmail.com (N.A.K.); grishin-a1@yandex.ru (A.V.G.); lenok27microb@gmail.com (E.P.M.); sofya_dadashyan@mail.ru (S.R.K.); lunin1955@gmail.com (V.G.L.); wowaniada@yandex.ru (V.A.G.); 2 All-Russia Research Institute of Agricultural Biotechnology, 127550 Moscow, Russia; 3 Faculty of Biology, MSU-BIT Shenzhen University, Shenzhen 518115, China; trusova.cat2017@yandex.ru; 4 Faculty of Biology, M.V. Lomonosov Moscow State University, 119234 Moscow, Russia; ant_bonar@mail.ru; 5 Research Center of Biotechnology of the Russian Academy of Sciences Leninsky Ave, 33, Bld. 2, 119071 Moscow, Russia; dudunandrey@mail.ru

**Keywords:** alginate gel, enzybiotics, endolysin, lysostaphin, biofilms, polymicrobial infection

## Abstract

The development of new and effective antibacterials for pharmaceutical or cosmetic skin care that have a low potential for the emergence and expansion of bacterial resistance is of high demand in scientific and applied research. Great hopes are placed on alternative agents such as bactericidal peptidoglycan hydrolases, depolymerases, etc. Enzybiotic-based preparations are being studied for the treatment of various infections and, among others, can be used as topical formulations and dressings with protein-polysaccharide complexes. Here, we investigate the antibiofilm properties of a novel enzybiotic cocktail of phage endolysin LysSi3 and bacteriocin lysostaphin, formulated in the alginate gel matrix and its ability to control the opportunistic skin-colonizing bacteria *Staphylococcus aureus*, *Pseudomonas aeruginosa*, and *Klebsiella pneumoniae*, as well as mixed-species biofilms. Our results propose that the application of SiL-gel affects different components of biofilm extracellular polymeric substances, disrupts the matrix, and eliminates the bacteria embedded in it. This composition is highly effective against biofilms composed of Gram-negative and Gram-positive species and does not possess significant cytotoxic effects. Our data form the basis for the development of antibacterial skin care products with a gentle but effective mode of action.

## 1. Introduction

A diverse and intricately balanced consortium of microorganisms living on human skin forms the barrier that protects the skin and soft tissues and plays an important role in human health. However, when the skin is damaged or the endocrine and immune systems are compromised, a disbalance can occur. Under these conditions, normally commensal bacteria can become pathogenic and can cause a variety of cutaneous diseases such as acne, inflammatory dermatosis, and rosacea and can impede wound healing. In the most adverse cases such as trophic ulceration, the formation of chronic wounds can ensue. Such wounds are constantly at risk of being reinfected by highly resistant microorganisms and can lead to limb amputation or patients’ death [1,2]. The spectrum of pathogens in skin infections is often dominated by commensal bacteria that can colonize the wound or skin defect in the form of multi-layered polymicrobial communities surrounded by a self-produced protective extracellular biofilm matrix, causing inflammation [3]. The most common bacterial causative agents of skin and wound infections are *Staphylococcus aureus*, *Staphylococcus epidermidis*, *Pseudomonas aeruginosa*, *Cutibacterium acnes*, *Enterobacteriaceae*, *Enterococcus* spp., *Acinetobacter baumannii*, and other facultative anaerobic species. While Staphylococcal skin and soft tissue infections prevail, they can also involve other bacterial species, in which case multi-layered polymicrobial biofilms can form, with the complex microenvironment that supports the survival of co-infecting microorganisms. The treatment of such infections with existing antimicrobial drugs and cosmetics is often ineffective due to the hampered access of antimicrobials in biofilms which necessitates the use of significantly increased drug concentrations.

Thus, topically applied cosmetics, over-the-counter products, and therapeutic antimicrobials should (a) act against a consortium of bacteria including aerobic and anaerobic multidrug-resistant microorganisms, (b) be effective against bacterial biofilms, and (c) limit their activity at the skin surface and soft tissues to prevent the development of antimicrobial resistance. These requirements are fully met by enzybiotics—enzyme-based antibacterials that include peptidoglycan hydrolases of phages and microorganisms which cause bacterial death through the cleavage of the chemical bonds within the cell wall peptidoglycan. The formulation of these bacteriolytic proteins into water-soluble gels is a promising approach, as this allows for the maintenance of the activity and the stability of the enzymes, provides the appropriate release rate, and modulates the inflammatory response and wound-healing process. For this purpose, natural biopolymers such as alginates could be applied. Alginates are widely used in biomedicine and cosmetics due to their high water-retention capacity (moisturizing properties), biosimilarity, and biodegradability. In addition, enzybiotic-based gels are not stable in the environment and quickly degrade when released into wastewater or the environment, thus limiting the selective pressure for the emergence of antimicrobial resistance.

The possibility of developing enzybiotic-based cosmetic or medicinal products for topical application is being actively explored. Such studies are mainly focused on the elimination of staphylococcal infections. Several peptidoglycan hydrolases have been investigated. For example, endolysin LysGH15, together with the anti-inflammatory flavonoid apigenin, was included in the Aquaphor gel for the treatment of wound infections [4]. A thermoresponsive gel containing a combination of the cysteine, histidine-dependent amidohydrolases/peptidases (or CHAPs) domain of the phage K endolysin (CHAP_K_) and lysostaphin [5] was fabricated to achieve the controlled release of the enzymes. A well-studied enzyme lysostaphin has been formulated into a polyethylene glycol gel for the treatment of implant-associated infections [6], chitosan-based antimicrobial gels [7], and nano-emulgel [8]. Moreover, Gladskin^®^ skincare cetomacrogel-based creams and a gel containing Staphefekt SA.100 recombinant phage endolysin for the specific treatment of *S. aureus* infections without affecting the commensal skin bacteria are available as an over-the-counter treatment in Europe [9].

Although these gel-based formulations were shown to be effective against bacterial biofilms, their potential to target polymicrobial infections has not been investigated. In the present work, the bacteriolytic composition containing phage endolysin LysSi3 and anti-staphylococcal bacteriocin lysostaphin was incapsulated into an algal alginate matrix, resulting in a SiL-gel preparation that is active against both Gram-negative and Gram-positive bacterial species. The gel’s antibacterial properties were studied in vitro against dual-species biofilms, demonstrating a high capacity of the gel to disrupt biofilms formed by multidrug-resistant *S. aureus*, *P. aeruginosa*, and *Klebsiella pneumoniae* and to significantly reorganize the biofilms’ matrix architecture.

Our studies performed with SiL-gel show that alginates are a suitable carrier base for bacteriolytic enzymes that do not reduce their effectiveness. SiL-gel can be used as a non-toxic, environmentally friendly product to control and correct the microbiological composition of skin colonizers, restoring the balance to support and engage the skin’s natural healing process.

## 2. Results and Discussion

### 2.1. Activity of Lysins and Their Combinations against Bacterial Isolates

Bacteriophages, bacteria, and other organisms can be the sources of peptidoglycan-degrading enzymes [10]. Often, these proteins do not possess a broad spectrum of action and are restricted to species or even specific strains. However, more data are emerging about lysins capable of eliminating several species of either Gram-positive or Gram-negative bacteria, which is determined, among other things, by the structural features of peptidoglycan [11]. Little information is available on enzymes equally efficient against both Gram-positive and Gram-negative pathogens. Thus, for the effective therapy of complex multispecies consortia, such as skin microbiomes, a combination of several enzymes with different activity spectra could be applied. To mimic the skin infection with multispecies bacterial biofilms, we selected two Gram-negative clinical isolates characterized by multiple antibiotic resistances, including a resistance to 3rd generation cephalosporins and aminoglycosides [12], and a Gram-positive *S. aureus* 73–14 isolate that is resistant towards beta-lactam antibiotics, including methicillin (MRSA strain), and is prone to producing strong biofilms.

The combination of enzymes investigated in this study consisted of two recombinant lysins. The first one was the bacteriocin lysostaphin (LST) produced by *Staphylococcus simulans*. Lysostaphin is an M23 zinc-dependent peptidase hydrolyzing glycyl-glycine peptide bonds in the cross-bridge of staphylococcal peptidoglycan [13] and is known to effectively kill *S. aureus* strains. The second lysin is a phage-derived endolysin LysSi3 that was previously characterized [14,15] as a family 24 glycoside hydrolase (muramidase) with significant antimicrobial and antibiofilm activity against *P. aeruginosa*, *A. baumannii*, *E. coli*, and some *K. pneumoniae* strains.

**Figure 1:**
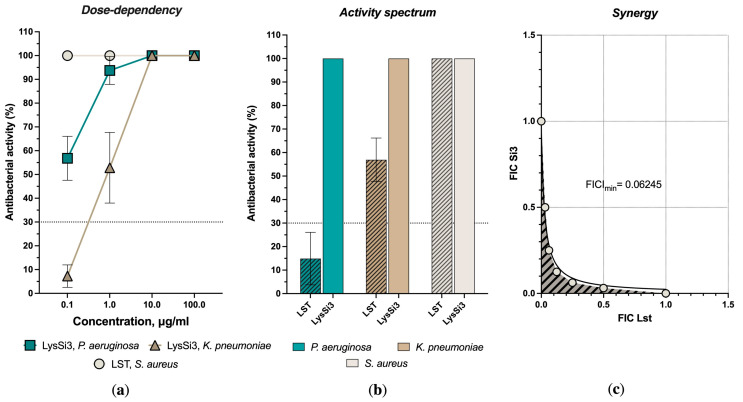
Antimicrobial activity of bacteriolytic enzymes against investigated strains. (**a**) Concentration-dependent antibacterial activity for LysSi3 (*K. pneumoniae* and *P. aeruginosa*) and LST (*S. aureus*). The threshold of 30% activity is indicated as a dashed line; (**b**) Bacterial reduction after the incubation with LysSi3 and LST in 100 μg/mL concentration, estimated by microplate assay. Hatched columns refer to LST; (**c**) Effects of two enzyme combinations on *S. aureus*. Minimum fractional inhibitory concentration index (FICI_min_) for combination is indicated. For all experiments, the mean values are shown from three independent experiments (±standard deviation, SD).

First, we evaluated the activity of the lytic enzymes against Gram-negative and Gram-positive clinical isolates using two in vitro methods: an antibacterial activity plate test and an estimation of the minimum inhibitory and bactericidal concentrations (MICs and MBCs, respectively). The plate test showed that LysSi3 acted on *K. pneumoniae* F 104-14 and *P. aeruginosa* Ts 38-16 in a dose-dependent manner and effectively eliminated bacteria at a 10 μg/mL concentration (Figure 1a). All the measured concentrations of LST were 100% effective against the *S. aureus* isolate.

An assessment of the activity spectra of enzymes at a high concentration (100 μg/mL) showed a 100% effectiveness of LysSi3 against all three investigated bacteria. LST possessed high bactericidal activity against *S. aureus* (Figure 1b) and a moderate effect on *K. pneumoniae* (about a two-fold reduction in bacterial growth compared to the buffer control). Thus, we have shown that, at high concentrations, both lysins demonstrate cross-specificity and are able to act on various species regardless of their Gram-staining status and the PG structure. We assume that the antimicrobial activity of these enzymes could be caused not only by the catalytic activity but also by other interactions of the enzymes with receptors of bacterial cells, for example, due to the membrane-permeabilizing ability of specific lysin domains [11,16,17].

We could estimate the MIC and MBC values for LST only, which, in the case of *S. aureus*, were 2.5 and 5 μg/mL, respectively. It was not possible to determine the MIC for LysSi3, although there was a pronounced tendency towards a two-fold decrease in the optical density of the bacterial suspensions of *K. pneumoniae* and *S. aureus* with an increase in the protein concentration of above 12.5–25 μg/mL. In general, the determination of the MIC using a standard protocol is often impossible for endolysins acting against Gram-negative pathogens despite the high antibacterial activity detected in other assays or in the presence of permeabilizing agents [18]. Previously, we obtained similar results for bacteriophage lysins, including the engineered endolysin LysECD7-SMAP [12], where a weak bactericidal activity was detected under the conditions supporting bacterial growth, accompanied by significant bactericidal activity when cultures were assayed in PBS or Tris-HCl buffers. However, when we assessed the activity of LST and LysSi3 in combination, a pronounced synergistic effect was observed in the MIC and MBC of *S. aureus*. While LysSi3 showed individual activity against this isolate only at high concentrations, when it was used in combination with LST, we observed a reduction in both MIC and MBC to less than 0.078 and 12.5 μg/mL for LST and LysSi3, respectively. Conversely, no synergy or additive effects of the two bacteriolytic enzymes were detected for Gram-negative strains.

The synergistic effects are well described for combinations of endolysins acting against Gram-negative species and cationic polypeptide antibiotics such as polymyxins [19,20]. These effects were explained by the additional destabilizing effect of peptides and enhanced endolysins’ penetration towards periplasmic peptidoglycan. However, no similar effects were previously described in the literature for combinations of peptidoglycan-degrading enzymes that are active against Gram-negative and Gram-positive pathogens. In the case of endopeptidase LST and muramidase LysSi3, synergy could be achieved by the hydrolysis of various bonds in PG and by the deeper and complete degradation of this polymer.

### 2.2. Antibiofilm Activity of Lysins and Their Combinations

All three bacterial isolates used in this study formed robust biofilms. Concentrations lower than 64 μg/mL of tetracycline, meropenem, and vancomycin (*S. aureus*) or polymyxin B (*P. aeruginosa*), had no effect on the 24–72 h biofilms, indicating a resistance of the biofilms towards the antibiotics that are active against the planktonic cells of these species.

**Figure 2:**
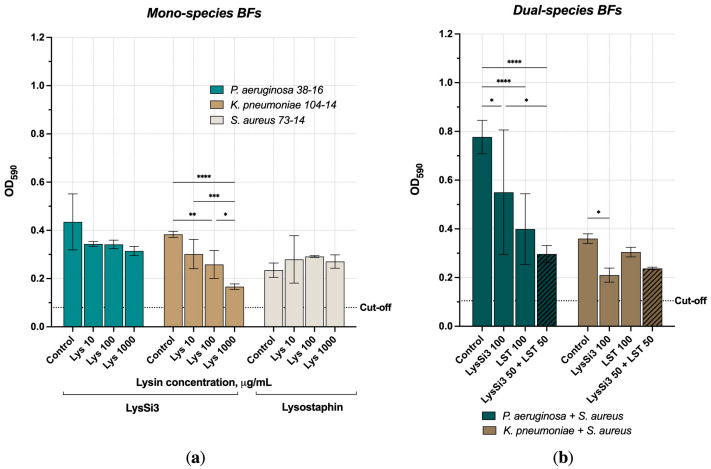
Antibiofilm activity of bacteriolytic enzymes and their combination against preformed bacterial films. (**a**) Mono-species bacterial biofilms. Lys—lysins. Cut-off indicating the background staining of wells containing a control broth is shown by the dashed line; (**b**) Biofilms formed by two bacterial species. Hatched columns indicate a combination of lysins. The concentrations of LysSi3 and LST (μg/mL) are given in row titles. Data are shown as mean ± SD. * — *p* < 0.033, ** — *p* < 0.002, *** — *p* < 0.0002, **** — *p* < 0.0001.

We assessed the ability of peptidoglycan hydrolases to degrade preformed mono- and dual-species biofilms alone and in combination. The effect of lysins varied with the species forming the biofilm (Figure 2a). Among the mono-species biofilms, the most pronounced antibiofilm effect was detected for *K. pneumoniae* treated with LysSi3. The biomass decreased 1.3–2.3-fold compared to the buffer-treated biofilm and depended on the concentration of the enzyme. The decrease in the *P. aeruginosa* biofilm biomass did not exceed 1.4-fold and was virtually independent from the endolysin concentration used. Surprisingly, no effect of LST was found for the staphylococcal mono-biofilm for all investigated LST concentrations.

When cultivating the bacterial mixtures (1:1), no significant changes in the biomass were observed for biofilms formed by *K. pneumoniae* and *S. aureus*, although the presence of both species was confirmed by quantitative PCR (qPCR). On the other hand, *P. aeruginosa* and *S. aureus* formed significantly more biomass compared to the mono-species biofilms (Figure 2b). This may be due to the adhesive properties of the *Pseudomonas* strain used in this study, since we detected low attachment levels of the *P. aeruginosa* Ts 38–16 biofilms to plastic and glass accompanied by a visible biomass growth. Many biofilm-forming staphylococci are non-motile, and, together with *P. aeruginosa*, dual-species biofilms could produce strong surface-associated communities embedded within an extracellular matrix. *P. aeruginosa* and *S. aureus* are among the most commonly associated microbial species in skin-wound infections [21].

As shown in Figure 2b, the use of a combination of lysins allowed for their concentration to be reduced while maintaining their effectiveness. In the case of *P. aeruginosa*, the effect of LysSi3 or LST individually at a concentration of 100 μg/mL reduced the biofilm biomass by 1.4–2.0 times, while their combination at 50 + 50 μg/mL effectively eliminated about 60% of the biofilm (a 2.6-time reduction compared to the control). Lysostaphin had a weak effect on the mixed *Klebsiella + Staphylococcus* biofilms but, in combination, the two lysins gave an effect of a 35% biomass reduction.

A number of studies have shown that LST successfully disrupts the biofilms formed by lysostaphin-sensitive *S. aureus* and *S. epidermidis* (in increased concentrations) strains but does not affect the biofilms of lysostaphin-resistant staphylococci or Gram-negative bacteria [22]. Although we did not observe a significant effect of LST on the mono-species *S. aureus* 73–14 biofilm, the use of LST in combination with LysSi3 allowed for a decrease in the amount of biomass of multispecies biofilms at lower concentrations than individual enzymes. Thus, our results demonstrate that peptidoglycan hydrolases possess antibiofilm activity, are able to act on preformed biofilms, and can be effectively combined to combat multispecies biofilms even in moderate doses.

### 2.3. Gel with Peptidoglycan Hydrolases Disrupts the Preformed Multispecies Biofilms

Based on the data obtained for the proteins in the solution, we encapsulated a composition of bacteriolytic enzymes into an alginate vehicle, resulting in an alginate antibacterial gel with 1 mg/mL of LysSi3 and 0.1 mg/mL of LST (SiL-gel) to target the multispecies biofilms of skin-colonizing pathogens. To assess the efficacy of this formulation, two types of biofilms were investigated: dual-species biofilms formed on plastic surfaces by *S. aureus* and either *K. pneumoniae* or *P. aeruginosa* as the Gram-negative component of the biofilm and dual-species bacterial biofilms grown on the human keratinocytes cell line (HaCaT) mimicking the in vivo tissue environment [23,24].

**Figure 3:**
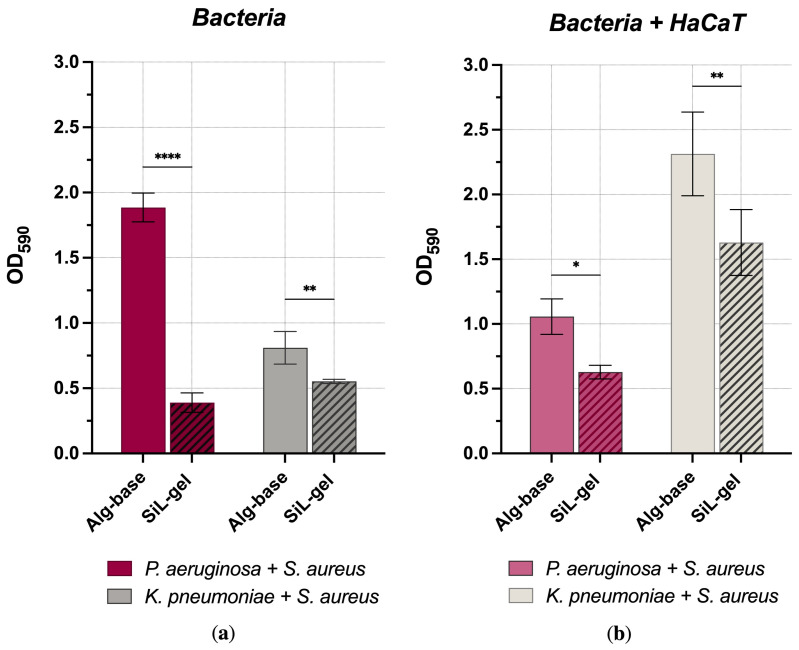
Antibiofilm activity of SiL-gel. (**a**) Dual-species bacterial films formed on plastic surfaces and incubated with samples for 2 h; (**b**) Biofilms formed by two bacterial species co-cultivated with HaCaT cells after a 5 h treatment with gel samples. Hatched columns indicate samples after incubation with lysin-containing gel. For all experiments, the mean values are shown from three independent experiments (±standard deviation, SD). * — *p* < 0.033, ** — *p* < 0.002, **** — *p* < 0.0001.

SiL-gel disrupted both types of biofilms. However, the effect varied depending on the species composition and the biofilm surface (Figure 3). The decrease in OD_590_ for the *K. pneumoniae* and *S. aureus* mixed biofilms was 1.4-fold compared to the Alg-base control both for the plastic-attached bacterial biofilms and the biofilms formed in the presence of a 3D host cell scaffold, despite the fact that, during the co-cultivation with HaCaT, the biofilm accumulated 2.5-times more biomass compared to the plastic-attached procaryotes biofilms.

Treatment of the *P. aeruginosa* and *S. aureus* mixed biofilms with SiL-gel reduced OD_590_ by 4.8-fold compared to the vehicle control (Alg-base), while treatment of the respective biofilms grown in the presence of eukaryotes had a more moderate effect and reached a 1.7-fold biomass reduction. Unlike the *Klebsiella + Staphylococcus* films, these types of combined biofilms grew better in the absence of host cell surfaces.

Thus, for the mixed *Klebsiella + Staphylococcus* biofilms, the action of both lysin mixture or SiL-gel was limited to 30–35% biofilm destruction and was largely indifferent to the concentration of lysins and the composition of the biofilm (the presence or absence of host cell surfaces). In the case of *P. aeruginosa* + *S. aureus*, biofilm elimination depended on the method of biofilm formation. In the absence of eukaryotic cells, a gel with lysins acted as effectively as a simple mixture of enzymes, achieving a dispersion of 80%. Only about half of the biomass was dispersed (40.5%) for biofilms grown in the presence of eukaryotes.

The peptidoglycan hydrolases used in this study are thermolabile water-soluble molecules that are supposed to be applied as pharmaceutical or cosmetic skin care formulations, including face or hairy skin area applications. Thus, the gel formulation can be considered as a favorable vehicle that is non-visible on the skin, spreadable, easy to use and that can dry on the skin surface, forming a greaseless non-occlusive film. Alginate biopolymers are non-toxic natural polysaccharides that are extensively used as a food and cosmetic additive with a great promise for their use as protein carriers [25,26]. They can form both gels and hydrogels and have been studied as injectable materials or a viscous vehicle for cosmetic applications, providing a controlled release of biologically active substances. Due to its wound-healing and gelling properties, alginate matrices are used as delivery systems to encapsulate hydrophilic active molecules.

Since alginates are polyanionic polymers, the electrostatic interactions comprise a significant driving force of the protein–polysaccharides complex formation. At the same time, peptidoglycan hydrolases are small proteins often possessing regions with the high positive charge density that can play a functional role in antimicrobial activity [16,17]. Although electrostatic interactions may increase alginate complexation with lysins, they can also affect the antimicrobial properties of the enzymes when encapsulated in a polymer matrix, and this should be monitored. Here, we demonstrated that the encapsulation of phage endolysin LysSi3 and bacteriocin LST in an alginate gel did not hinder the enzymes’ effects. The application of SiL-gel is highly effective against mixed biofilms composed of *P. aeruginosa* and *S. aureus* and is also active, albeit to a lesser extent, against biofilms with *K. pneumoniae* as the Gram-negative component.

### 2.4. Cytotoxicity of Alginate Gel with Bacteriolytic Enzymes

To evaluate the cytotoxic effects of enzymes incapsulated in an alginate in different concentrations (ranged from 17.2 µg/mL to 2200 µg/mL), the Murine NIH 3T3 fibroblasts were chosen.

**Figure 4:**
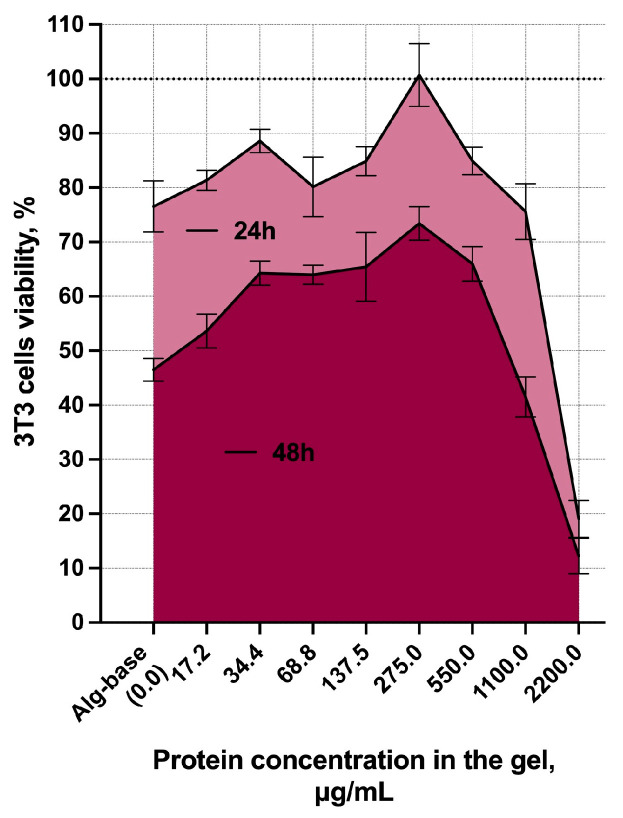
Percentage of living cells after 24 h and 48 h of incubation with SiL-gel-containing enzymes at different concentrations. The mean values with SD are shown, and all experiments were performed in five replicates.

The addition of SiL-gel has a small cytotoxicity effect [27] on cell growth after 24 h (around 30%) followed by 50% cell viability after a 48 h incubation in almost all investigated concentrations of enzymes (Figure 4).

The alginate base itself showed 76.5 and 46.5% cell viability during 24 h and 48 h, respectively. These results indicate that a cocktail of peptidoglycan hydrolases significantly affects the eukaryotic cells growth at concentrations higher than 1000 µg/mL and reduces the rate of viable cells to less than 20% in a 2200 µg/mL concentration. The decrease in cell number after 48 h of cultivation in the presence of sodium alginate can be explained by the high viscosity of the preparation, resulting in a low nutrient and oxygen availability to the cells during the cultivation. Under in vivo system conditions, the results can differ significantly since the availability of nutrients for cells is ensured by their microenvironment.

Thus, the cell model used in this study should be optimized to investigate the cytotoxic effects of the obtained formulation; however, it showed that SiL-gel preliminarily has an acceptable in vitro safety profile and does not significantly suppress cell growth.

### 2.5. Modification of Biofilms’ Matrix Architecture under Gel with Peptidoglycan Hydrolases

To assess the effect of SiL-gel on the composition and structure of bacterial biofilms, a microscopic investigation of the *P. aeruginosa* and *S. aureus* mixed cultures grown on slides was performed. We used several dyes for the identification of different components of the biofilm, including crystal violet (CV)—a commonly used biofilm dye which unspecifically binds to polyanionic biopolymers—the FilmTracer™ SYPRO™ Ruby fluorescent dye was used to label the extracellular polymeric matrix of biofilms and the different classes of the proteins within them, and 4′,6-diamidino-2-phenylindole (DAPI) was used to visualize the extracellular DNA (eDNA) in living biofilms [28]. Here, we used a short incubation with the DAPI dye, which prevents the cell penetration and staining of intracellular DNA but allows for good binding to the eDNA of the biofilm matrix [29]. Furthermore, the abundance of live and dead cells within the matrix was investigated.

**Figure 5:**
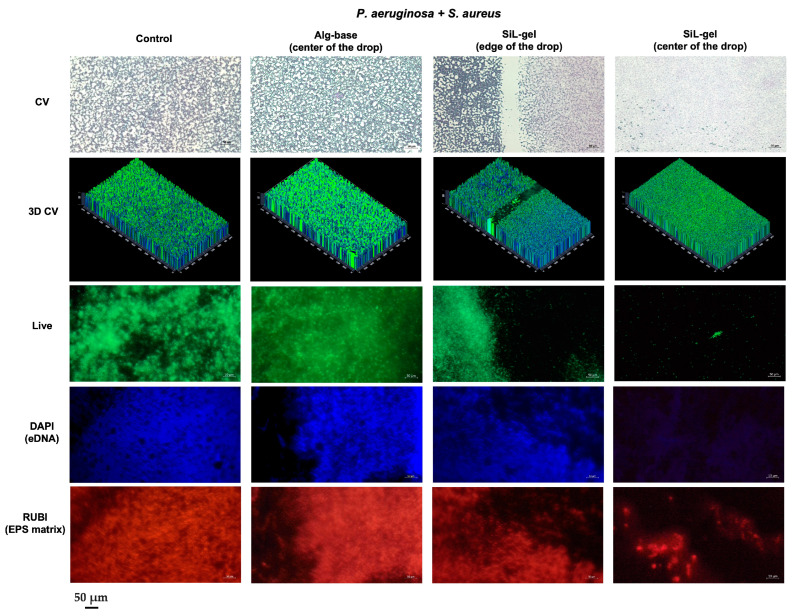
Microphotographs of non-treated (control) or treated with Alg-base or SiL-gel biofilms formed on a glass by *S. aureus* and *P. aeruginosa*. Slides were stained with CV—crystal violet—the FilmTracer™ LIVE/DEAD^®^ Biofilm Viability Kit, DAPI Nucleic Acid Stain, and FilmTracer™ SYPRO^®^ Ruby Biofilm Matrix Stain and imaged at ×400 and ×630 magnification. Three-dimensional modeling of CV-stained biofilms was generated with the ZEN 3.0 software (Zeiss AG).

Crystal violet staining of the mixed-species biofilms showed intensively stained cell aggregates of different sizes on the non-treated glass slides, which did not change their integrity after Alg-base application (Figure 5). On the contrary, incubation with SiL-gel resulted in the formation of a well-defined boundary and the absence of a three-dimensional biofilm structure in the center of the SiL-gel drop. A faint crystal violet staining was detected on the glass surface of the slides which was attributed to the previous localization of the attached biofilms.

Changes in the biofilm extracellular polymeric substance (EPS) consisting of numerous polysaccharides, nucleic acids, and proteins that provide stability to the biofilm and microenvironment for the bacteria were studied with fluorescent microscopy. As revealed by the PCR analysis, the *P. aeruginosa* 38–16 strain has a genetic background for the synthesis of all three biofilm exopolysaccharides (see Section 2.6), which are the integral components of the extracellular biofilm matrix: Psl, Pel, and alginate—neutral, cationic, and anionic polysaccharides, respectively. Thus, biofilms formed by the investigated species may include a wide range of different pseudomonal and staphylococcal polysaccharides.

Our experiments indicated that the disruption of the EPS was quite uniform upon SiL-gel application. Visualization of the protein and glycoprotein components of the EPS matrix by the FilmTracer™ dye revealed a presumably unspecific matrix dispersion by the SiL-gel. Although some stained compounds were still observed after incubation with the gel, these structures were strongly reduced and dispersed compared to the Alg-base samples.

We also observed a significant amount of eDNA within the biofilm that is known to promote resistance to highly charged antimicrobials through electrostatic interactions, neutralizing the charge and limiting their mobility. However, after SiL-gel treatment, structures containing eDNA were destabilized to a large extent, leaving weak fluorescent traces at the site of previously attached biofilms, similar to the CV staining. The loss of nucleic acid compounds can also affect the biofilm architecture, breaking the interactions of eDNA with biofilm proteins and polysaccharides [30].

Fluorescent staining with the viability kit resulted in a prominent detection of live bacterial cells with intact membranes, the amount of which was drastically reduced on the SiL-gel-treated slides compared to the Alg-base and non-treated samples. However, since the dye used for this assay is the SYTO^®^ 9 permeant green fluorescent nucleic acid stain, we suppose that the eDNA content of the biofilm could be visualized by this method as well. On the contrary, dead bacterial cells stained with a non-permeant red fluorescent nucleic acid stain propidium iodide were not detected on any of the slides. However, considering the poor integrity of the cells treated with lysins [14] and a washing step performed before the labeling, we did not expect significant staining in this case.

To sum up, the microscopic investigation of SiL-gel action on biofilms preformed on glass slides demonstrated that the combination of two peptidoglycan hydrolases in the alginate gel led to bacteria elimination in the dual-species biofilms, as well as a weakening and reorganization of the biofilm architecture. Although the exact chemical and physical composition of the EPS varies between species and growth conditions, the matrix provides mechanical stability and creates compartmentalized chemical and physical microenvironments that afford protection of the cells within a heterogeneous 3D structure [31]. Biofilms formed in clinical specimens tend to consist of cellular aggregates of varying sizes and shapes that are largely dependent on biofilm maturity and qualitative composition [32]. The breakdown of these structures is a fundamental step for the effective action of antimicrobials. A disruption of the viscous properties of the matrix can further reduce biofilm cohesion, thus enhancing enzymes’ antimicrobial efficacy. The peptidoglycan hydrolases initially developed to target bacterial cells turned out to possess two modes of action: cell bactericidal activity and matrix dispersion. These properties make peptidoglycan hydrolases more attractive compared to EPS-degrading enzymes (e.g., glucano-hydrolases or DNAses) that can be used only as an adjunctive approach for biofilm control. Additionally, targeting matrix components allows for the evasion of the interaction with the intracellular mechanisms of resistance and for the slowing down of the resistance emergence.

### 2.6. SiL-Gel Affects the Expression of Genes Related to the Metabolism and Maintenance of Biofilm Architecture

We also measured the relative expression of several genes regulating the synthesis, attachment, and maintenance of biofilms in the investigated bacteria to assess the impact of SiL-gel. For *P. aeruginosa*, the genes related to the metabolism of three crucial exopolysaccharide alginates, (*algD*), Psl (*pslA*), and Pel (*pelF*), were chosen. AlgD plays a key role in the synthesis of the alginate precursor, GDP-mannuronic acid, and the control of alginate synthesis and regulates the transcription of Alg proteins [33]. Psl and Pel exopolysaccharides facilitate initial surface attachment and help to form and maintain the biofilm architecture [34,35,36,37]. For *K. pneumoniae*, the genes linked to EPS production (*pgaA*) and type 3 fimbriae (*mrkA*) were used. These genes are associated with the ability to produce a rich biofilm and to determine its adherence properties [38]. For *S. aureus*, we selected the genes from intracellular adhesin operon (*icaA-icaR*) that are associated with polysaccharide intracellular adhesins (PIAs) and capsular polysaccharide adhesin (PS/A) production and that are responsible for biofilm metabolism [39]. The presence of all biofilm-related genes in the studied strains was preliminarily confirmed.

**Figure 6:**
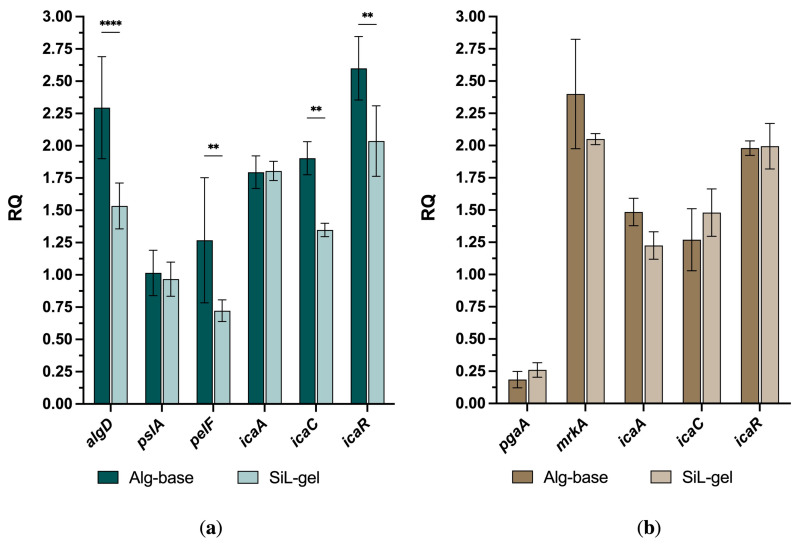
BF-related gene expressions in biofilms that were treated with the alginate vehicle (Alg-base) and SiL-gel. (**a**) *P. aeruginosa* and *S. aureus* dual-species; (**b**) *K. pneumoniae* and *S. aureus* dual-species. For all experiments, the mean values are shown from three independent experiments (±standard deviation, SD). ** — *p* < 0.002, **** — *p* < 0.0001.

After biofilm incubation with SiL-gel, most of the genes were downregulated (Figure 6), which was especially pronounced for the mixed biofilms containing *P. aeruginosa*. The relative expression of the *algD*, *pelF*, *icaC*, and *icaR* genes was 1.3–1.8 times lower compared to the RQ in the *P. aeruginosa* and *S. aureus* dual-species biofilms that were treated with the alginate base. The *pslA* and *icaA* gene expressions remained at levels of the Alg-base samples. No statistically significant changes in gene expression were observed in the *K. pneumonia* and *S. aureus* dual-species biofilms, with the relative quantity of *pgaA*, *icaC*, and *icaR* remaining stable in all the measured samples (0.9–1-fold changes), and the *mrkA* and *icaA* genes were slightly downregulated (about 1.2-fold).

Although the antibiofilm activity of lysins is primarily associated with the catalytic and non-enzymatic destruction of bacterial cells and the biofilm matrix, we also detected a negative effect on the expression of the genes regulating the synthesis of exopolysaccharides that are crucial for biofilm formation. We attribute this effect to the indirect action of products formed during the biofilm degradation (e.g., polysaccharides derivatives), affecting the biosynthetic pathways within the bacterial community and promoting the SiL-gel antibiofilm activity. However, the mechanism underlying the observed effects is a matter of further study.

## 3. Conclusions

Previously, attempts have been made to develop therapeutics against infections caused by *S. aureus* using alginate hydrogels loaded with enzybiotics or enzybiotic-derived peptides [40]. Gels with Gram-negative-acting endolysins are also considered as perspective topical treatment options [41]. However, alginate-based compositions with bacteriolytic enzymes have not been described as cosmetic formulations. We demonstrated that the combination of phage endolysin LysSi3 and bacteriocin LST encapsulated in an alginate gel could be efficiently applied to treat mixed biofilms composed of both Gram-negative and Gram-positive bacteria without a significant loss of antimicrobial activity or the acquisition of cytotoxic properties after encapsulation. Moreover, such a preparation possesses both a direct effect on biofilms throughout the dispersion of the matrix structure and an elimination of the cells contained within it and is also capable of triggering mediated processes of biofilm destruction, including those at the gene-expression level.

To sum up, our study highlights previously unreported characteristics of an alginate gel loaded with a novel enzybiotic cocktail that can be fabricated as cosmetic and medical skin care formulations and dressings with a gentle but effective mode of action.

## 4. Materials and Methods

### 4.1. Bacterial Strains Used in this Study

The bacterial strains used in this study include multidrug-resistant clinical isolates of Gram-negative (*P. aeruginosa* 38–16 isolate that is resistant to ampicillin, tetracycline, chloramphenicol, cefotaxime, ceftazidime, gentamicin, meropenem, and cefoperazone/sulbactam; and *K. pneumoniae* 104–14 isolate that is resistant to ampicillin, chloramphenicol, cefotaxime, ceftazidime, gentamicin, ertapenem, imipenem, and meropenem) and Gram-positive (*S. aureus* 73–14, a methicillin-resistant strain) pathogens from the collection of the Gamaleya National Center for Epidemiology and Microbiology. All the strains were stored at −80 °C and were cultivated in the appropriate medium at 37 °C and 250 rpm overnight before performing the assays.

### 4.2. Proteins

Recombinant endolysin LysSi3 was obtained as previously described [14] in the *Escherichia coli* strain BL21(DE3) pLysS using immobilized-metal affinity chromatography purification. LST recombinant expression and purification is described in detail in [42]. Proteins were lyophilized and stored at −80 °C until the experiments.

### 4.3. Antibacterial In Vitro Activity Test

Overnight bacterial cultures (OD_600_ of 1.4–1.6) that were cultivated in a Mueller–Hinton broth (MHB) were diluted with sterile MHB and were grown to the OD_600_ of 0.6 a.u. Bacteria were harvested at 3000× *g* for 10 min and were resuspended in a PBS buffer (pH 7.4) to a concentration of ~1.5 × 10^8^ CFU/mL, corresponding to the 0.5 McFarland units. The bacterial suspensions were 100-fold diluted with the 20 mM Tris-HCl buffer (pH 7.5), so the final density was approximately 10^6^ CFU/mL. Afterwards, the bacterial suspension and the proteins (LysSi3 or LST), in appropriate concentrations, were mixed 1:1 in volumes of 100 μL in 96-well plates. Samples containing the 20 mM Tris-HCl buffer (pH 7.5) were used as a negative control. Samples were incubated at 37 °C and 200 rpm for 2 h. Subsequently, 100 μL of 10-fold PBS dilutions were plated onto an LB agar and incubated overnight at 37 °C, and the number of bacterial colonies was counted. The experiments were performed in triplicate, and the antibacterial activity was estimated as follows:

(1) $$Antibacterial\;activity\left(\%\right)=100-\frac{CFUexp}{CFUcont}\times 100,$$

where CFUexp is the CFU number in the experimental culture plates, and CFUcont is the CFU number in the control culture plates. Antibacterial activity was regarded as meaningful when it exceeded 30%.

The dose-dependent antibacterial activity of LysSi3 was investigated using Gram-negative *K. pneumoniae* and *P. aeruginosa* isolates, while *S. aureus* was used for LST. The protein concentrations investigated in this assay were 0.1 to 100 μg/mL of each protein. For the activity spectrum analysis, each of enzymes was assayed using all three bacterial strains with the final protein concentration of 100 μg/mL.

### 4.4. Enzyme Synergy Assay

We used a checkerboard assay based on the serial microdilution method to evaluate the effectiveness of the combinations of endolysins against Gram-negative and Gram-positive bacteria [19,43]. Two-fold dilutions of the LysSi3 (12.5–200 μg/mL) and LST (0.078–10 μg/mL) for the Gram-positive strain were prepared in the 20 mM Tris-HCl buffer (pH 7.5). For the Gram-negative strains, the investigated concentrations of both LysSi3 and LST were 12.5–100 μg/mL.

The isolates were grown on a Mueller–Hinton agar, and up to 5 individual bacterial colonies were resuspended in PBS (pH 7.4) to a concentration of ~1.5 × 10^8^ CFU/mL, according to the 0.5 McFarland turbidity standard, following a 100-fold dilution with MHB to a concentration of 10^6^ CFU/mL. Further, 50 μL of bacterial suspensions were incubated with bacteriolytic enzymes at 37 °C without aeration for 16–20 h, and MICs were detected by OD_600_ measurement and were visually confirmed. The fractional inhibitory concentrations (FICs) for both proteins were calculated by dividing the MIC of two enzymes in combination with the MIC of each enzyme alone. For each combination, the fractional inhibitory concentration index (FICI) was calculated, which represents the sum of the LysSi3 and LST FICs. The effects were evaluated according to resulted FICI for each combination: 0 < x ≤ 0.5—synergism; 0.5 < x < 1—partial synergism; 1 ≤ x ≤4—indifference; x > 4—antagonism.

Additionally, for the protein combinations against *S. aureus*, an MBC value was estimated by plating mixtures without visible bacterial growth onto an MHB agar, followed by an overnight incubation at 37 °C, accounting for the CFU number.

### 4.5. Mono- and Dual-Species Biofilm Formation

#### 4.5.1. Bacterial Biofilm Formation

Overnight bacterial cultures grown in a Tryptic Soy Broth (TSB) (BD, Franklin Lakes, NJ, USA) were harvested (6000× g, 5 min, RT) and resuspended in PBS (pH 7.4) to a concentration of ~3 × 10^8^ CFU/mL (1.0 McFarland units). Each bacterial suspension was 100-fold diluted in the TSB to a final density of approximately 10^6^ CFU/mL. To allow for mono-species biofilm formation, 100 μL of the bacterial suspension was added to the wells of 96-well sterile polystyrene cell culture plates (Eppendorf, Hamburg, Germany) and was incubated for 48 h at 37 °C and 250 rpm for *K. pneumoniae* and *S. aureus* or for 24 h at 37 °C without aeration for *P. aeruginosa*.

To obtain the dual-species biofilm suspensions of both bacterial strains (*Pseudomonas*-*Staphylococcus* or *Klebsiella*-*Staphylococcus*), they were mixed in 1:1 (*v/v*) ratio and incubated as *Klebsiella*- or *Pseudomonas*-containing cultures.

#### 4.5.2. Bacterial Biofilm Co-Cultivated with HaCaT Cells

HaCaT cells (3 × 10^4^ cells/well) were seeded in 96-well tissue culture-treated plates (Eppendorf, Hamburg, Germany) and were cultivated in 100 μL of a DMEM medium (PanEco, Moscow, Russia) supplemented with a 10% fetal bovine serum (FBS) (Citiva, FL, USA), 1% glutamine without antibiotics for 24 h in a humidified atmosphere of 5% CO_2_, and 95% air at 37 °C.

Overnight bacterial cultures in TSB were harvested (6000× *g*, 5 min, RT) and resuspended in PBS (pH 7.4) to a concentration of ~1.5 × 10^8^ CFU/mL (0.5 McFarland units). Each bacterial suspension was 1000-fold diluted in DMEM to a final density of approximately 10^5^ cells/mL. Following a 24 h HaCaT cell incubation, the medium was removed, and 100 μL of prepared dual-species (1:1, *v/v*) bacterial suspensions in DMEM were added into each well and incubated for 18–20 h.

### 4.6. Gel Formulation

A 2% solution of algae sodium alginate (AppliChem, Darmstadt, Germany) in a sterile 20 mM Tris-HCl (pH 7.5) was prepared by mixing until complete dissolution and was sterilized at 121 °C and 1 atm for 15 min. After gel cooling, sterile 20 mM Tris-HCl (pH 7.5) was aseptically added in equal proportions to obtain the 1% alginate vehicle, or filter-sterilized LysSi3 to a final concentration of 1 mg/mL and LST to a final concentration of 0.1 mg/mL were added and mixed thoroughly to obtain the SiL-gel.

### 4.7. Efficacy of Bacteriolytic Enzymes and Antibacterial Gel

#### 4.7.1. Antibiofilm Activity Assessment

Microplate wells containing the preformed biofilms (Section 4.5.1) were washed with 200 μL of PBS (pH 7.4) for three times and were air-dried for approximately 15 min. Then, 100 μL of individual enzymes solutions (10, 100, or 1000 µg/mL) for the mono-species biofilms, and 100 µg/mL of the individual enzyme solutions or their 1:1 (*v/v*) mixtures for the dual-species biofilms were added to the wells and were incubated at 37 °C and 250 rpm for 2 h. The Tris-HCl buffer (20 mM, pH 7.5) was used as a negative control.

For the assessment of the antibiofilm activity of Sil-gel, dual-species biofilms formed in microplates alone (Section 4.5.1) or co-cultivated with HaCaT cells (Section 4.5.2) were treated with SiL-gel (LST 0.1 mg/mL and LysSi3 1 mg/mL) or the alginate base as a negative control at 37 °C and 250 rpm for 2–5 h. After incubation, wells were carefully washed twice with 200 µL of PBS (pH 7.4) and were air-dried. Then, biofilms were stained with 100 μL of 0.1% crystal violet (Applichem, Darmstadt, Germany) for 20 min, followed by triple rinsing with water. The remaining stain was re-solubilized in 200 µL of 33% acetic acid, and the OD_590_ of the obtained solution was measured using the SPECTROstar NANO Absorbance Reader (BMG LABTECH, Ortenberg, Germany). All experiments were performed in quintuple. The biofilm formation interpretation was performed according to [44].

#### 4.7.2. Microscopy

For microscopy, dual-species biofilms (*Pseudomonas*-*Staphylococcus*) were preformed on a glass. Sterile glass coverslips (Hampton Research, Aliso Viejo, CA, USA) were plunged in Petri dishes containing the bacterial cultures mixture 1:1 (*v/v*) in TSB with approximately 10^6^ CFU/mL of each strain. The dishes were incubated at 37 °C for 48 h without shaking. Slides were carefully washed with sterile water, and then, 10 μL of SiL-gel or the vehicle gel (Alg-base) was dropped onto the slides, and they were incubated for 2 h at room temperature. Afterward, the slides were again carefully washed with sterile water two times.

Air-dried slides were stained with a 0.1% crystal violet solution for 15 min at RT, rinsed with water, and air-dried for bright-field microscopy. Other slides were stained with the DAPI Nucleic Acid Stain (Molecular Probes, Invitrogen, Eugene, OR, USA), the FilmTracer™ SYPRO^®^ Ruby Biofilm Matrix Stain (Molecular Probes, Invitrogen, Eugene, OR, USA), and the FilmTracer™ LIVE/DEAD^®^ Biofilm Viability Kit (Molecular Probes, Invitrogen, Eugene, OR, USA), according to the manufacturers’ instructions for dark-field fluorescent microscopy. All slides were imaged with the Axiostar Plus Transmitted Light Microscope (Zeiss AG, Jena, Germany) at ×400 and ×630 magnification. Microphotographs were proceeded with the ZEN 3.0 (blue edition) software (Zeiss AG, Jena, Germany).

#### 4.7.3. Nucleic Acid Isolation and qRT-PCR of BF-Related Genes

The dual-species biofilms were cultured as described in Section 4.5.1. and were treated with a 20 mM Tris-HCl (pH 7.5) buffer, a vehicle gel (Alg-base), or a lysin-containing gel (SiL-gel) for 2 h (see Section 4.7.1). The microplate wells were twice washed with PBS.

The total RNA was extracted from biofilm lysates using the Extract RNA Reagent (Eurogen, Moscow, Russia), following the manufacturer’s instructions. The amplification and quantification of bacterial RNA were carried out by using a one-step RT-qPCR technique. The reaction mixture containing (for one reaction) 5 pmol of each primer, 3 pmol of the probe, 12.5 μL of the 2X BioMaster RT-PCR (Biolabmix, Moscow, Russia), and 10 μL of the RNA was used. The primers and probes were designed to target genes related to biofilm formation in the investigated species (gene of interest, GOI). The total volume of one reaction mixture was 25 μL. Amplification was performed using a Real-Time CFX96 Touch instrument (Bio-Rad, USA). The conditions of the one-step RT-qPCR reaction were as follows: 50 °C for 15 min and 95 °C for 5 min, followed by 45 cycles of 95 °C for 10 s and 55 °C for 1 min.

The 2^−ΔCt^ method was used to analyze the relative gene expression based on the melting curve. The *gmk*, *rpsL*, and *proC* were considered as the housekeeping genes (HKs) to determine the expression level of the genes related to the biofilm formation of *S. aureus*, *P. aeruginosa*, and *K. pneumoniae*, respectively. The fold change in the gene expression was determined relative to the housekeeping genes (HKs) and was validated using data analysis tools. The list of primers and probes is provided in Table 1.

### 4.8. Cytotoxicity Test for Alginate Matrices with the Addition of Lysins

The cytotoxic properties were evaluated for the 3T3 fibroblast cell line using samples of a 1% algae sodium alginate solution with LysSi3 and LST enzymes in a full growth medium (DMEM) (Biosharp, Hefei, China), supplemented with 10% FBS (Biosharp, Hefei, China) and 1% of an antibiotic (antibiotic–antimycotic, Thermofisher scientific, Waltham, MA, USA).

The 3T3 cells were added to a 96-well plate at 10,000 cells per well and were cultivated in 200 µL of a growth medium over 24 h at 37 °C in a humidified atmosphere with 5% CO_2_, until 80–90% of a confluent monolayer was achieved. After incubation, 25 µL of the 1% sodium alginate solution with lysins was added to the cells. The final volume in each well was 125 µL. The final protein (LysSi3 + LST) concentrations in the wells were 2200, 1100, 550, 275, 137.5, 68.8, 34.4, and 17.2 µg/mL. A total of 25 µL of the samples of the sodium alginate solution without enzymes was used as a control of the alginate effect. Cells cultivated in 125 µL of the growth medium were used as a positive control and were taken as a 100% cell viability for data analysis. Each sample type was analyzed in five replicates for statistical sampling.

Cytotoxicity of the samples was assessed using CCK-8 (Cell Counting Kit 8 (WST-8), Keygen Biotech, Nanjing, China). The plate was incubated at 37 °C with 5% CO_2_ for 24 and 48 h. Before staining, wells were washed from polymeric substances with PBS. Then, 10 µL of the WST-8 dye and 90 µL of the DMEM growth medium were added to each well. The mixtures were incubated for 2.5 h at 37 °C with 5% CO_2_. The liquid was transferred to another plate, and the absorbance, at a wavelength of 450 nm (OD_450_), was measured using a plate spectrophotometer.

Cell viability was calculated using the following equation:

(2) $$LC=\frac{Y_{Abs}-Y_{620}}{Y_{max}-Y_{620}}\times 100\%,$$

LC—percent of living cells in the well, %; Y_Abs_—value of OD_450_ in the well with a studied sample, a.u.; Y_max_—average value of the OD_450_ of the positive control, a.u.; Y_620_—value of the OD_620_ in the corresponding well with a studied sample, a.u.

### 4.9. Statistical Analysis

For the activity experiments, a two-way ANOVA with Tukey’s multiple comparisons test was used. To identify significant differences in the cytotoxicity test, Q-Dixon and T-Student criteria were used. Values of *p* < 0.05 were considered statistically significant. The data were analyzed using the GraphPad Prism 10.0 software.

## Figures and Tables

**Table 1:** Sets of primers and probes targeting genes related to biofilm formation.

Target Gene	Primers and Probes	
*Staphylococcus aureus*		
*icaA* (GOI)	Staur_icaA-F Staur_icaA-R Staur_icaA-P	5′-TAG AAT ATG CAA GTT TAA TTG GCT GTA-3′ 5′-TCA GTA TCC CAG TAG CCA AC-3′ 5′-R6G-CGA AGT CAG ACA CTT GCT GGC GCA GT-BHQ1-3′
*icaC* (GOI)	Staur_icaC-F Staur_icaC-R Staur_icaC-P	5′-GTT ATT ATG ATT GTA TTA GCT GTA GC-3′ 5′-CCC AAG ATA ACA ATA AAC ATA ATA CTA TT-3′ 5′-R6G-ATG GAG ACT ATT GGA ACG TTA CCA GCT T-BHQ1-3′
*icaR* (GOI)	Staur_IcaR-F Staur_IcaR-R Staur_IcaR-P	5′-TTC GAG TCA AAA TGG TAA TAT AAA CT-3′ 5′-TAA CGC AAT AAC CTT ATT TTC AGA G-3′ 5′-R6G-TCA TCA AGT GTT GTA CCG TCA TAC CCC-BHQ1-3′
*gmk* (HK)	Staur_gmk-F Staur_gmk-R Staur_gmk-P	5′-ATA AGT ATT CTA TTT CAA TGA CAA CAC G-3′ 5′-ATT AAA GCT TCA AAC GCA TCC CT-3′ 5′-FAM-CGT GAA GGT GAA GTT GAT GGC GTA GAT T-BHQ1-3′
*Pseudomonas aeruginosa*		
*algD* (GOI)	Psaer_algD-F Psaer_algD-R Psaer_algD-P	5′-GAC CAC AAG CTC AAC CTG TC-3′ 5′-CCT GGT TGG AGT TGC TGC-3′ 5′-R6G-CGC CCT CAC CTA TCG CGC CAG CCA-BHQ1-3′
*pslA* (GOI)	Psaer_pslA-F Psaer_pslA-R Psaer_pslA-P	5′-CTT GGT CAG GAG TAC GGC T-3′ 5′-CCC TGC ACA AGA TCA AGA AAC-3′ 5′-R6G-AGG ATG TAG AGG TCG AAC CAC ACC G-BHQ1-3′
*pelF* (GOI)	Psaer_pelF-F Psaer_pelF-R Psaer_pelF-P	5′-CAG GAC AAG GTG AAG TTC CT-3′ 5′-GAG GAT CAC CAG CGG CTG-3′ 5′-R6G-CTC GGC CTG ATG GTC CTC ACC TCG A-BHQ1-3′
*rpsL* (HK)	Psaer_ rpsL-F Psaer_ rpsL-R Psaer_ rpsL-P	5′-ATG GCA ACT ATC AAC CAG C-3′ 5′-ACG ACG TTG CGG GCA GTT-3′ 5′-FAM-CGC AAG CGC ATG GTC GAC AAG AGC-BHQ1-3′
*Klebsiella pneumoniae*		
*pgaA* (GOI)	Kpn_pgaA-F Kpn_pgaA-R Kpn_pgaA-P	5′-CCA GCT AAA AGC GGC GGA 5′-CAT TTG CCG CCA CTC CTG 5′-R6G-AGC CTG CAG CTG GAG CGC CA-BHQ1-3′
*MrkA* (GOI)	Kpn_MrkA-F Kpn_MrkA-R Kpn_MrkA-P	5′-ACG TCT CTA ACT GCC AGG C 5′-TAG CCC TGT TGT TTG CTG GT 5′-R6G-CTG GAC CGG CGG TAA CCT GCT G-BHQ1-3′
*proC* (HK)	Kpn_proC-F Kpn_proC-R Kpn_proC-P	5′-GAT TGC CGA TAT CGT CTT CG 5′-GAG ACC ACC AGC GAC TCT 5′-FAM-TCA TGA CCA AAG TGC TGG GCG ATA TC-BHQ1-3′

## Data Availability

The data presented in this study are openly available in the article.
